# Supplementary material for: Multi-feature clustering of CTCF binding creates robustness for loop extrusion blocking and Topologically Associating Domain boundaries
Source: Nat Commun. 2023 Sep 12;14:5615. doi: 10.1038/s41467-023-41265-y (PMC10497529; doi:10.1038/s41467-023-41265-y)
Supplement: Supplementary file 1 — Supplementary Information [file 41467_2023_41265_MOESM1_ESM.pdf]

Supplementary Information File

Multi-feature clustering of CTCF binding provides robustness for loop extrusion blocking and Topologically Associating Domain boundaries

---

Li-Hsin Chang[1,6*], Sourav Ghosh[1,7*], Andrea Papale[2], Jennifer M. Luppino[3], Mélanie Miranda[1], Vincent Piras[1], Jéril Degrouard[4], Joanne Edouard[1], Mallory Poncelet[1], Nathan Lecouvreur[1], Sébastien Bloyer[1], Amélie Leforestier[4], Eric F. Joyce[3], David Holcman[2,5], Daan Noordermeer[1#]

1 Université Paris-Saclay, CEA, CNRS, Institute for Integrative Biology of the Cell (I2BC), 91198, Gif-sur-Yvette, France
2 École Normale Supérieure, IBENS, Université PSL, 75005, Paris, France
3 Department of Genetics, Penn Epigenetics Institute, Perelman School of Medicine, University of Pennsylvania, Philadelphia, PA, USA
4 Université Paris-Saclay, CNRS, Laboratoire de Physique des Solides (LPS), 91405, Orsay, France
5 Churchill College, University of Cambridge, CB30DS, Cambridge, UK
6 Current affiliation: MRC Molecular Haematology Unit, MRC Weatherall Institute of Molecular Medicine, University of Oxford, and National Institute of Health Research, Blood and Transplant Research Unit in Precision Cellular Therapeutics, OX39DS, Oxford, UK
7 Current affiliation: Department of Pathology and Laboratory Medicine, Western University, N6A3K7, London, Ontario, Canada

* Equal author contribution
\# Correspondence: daan.noordermeer@i2bc.paris-saclay.fr

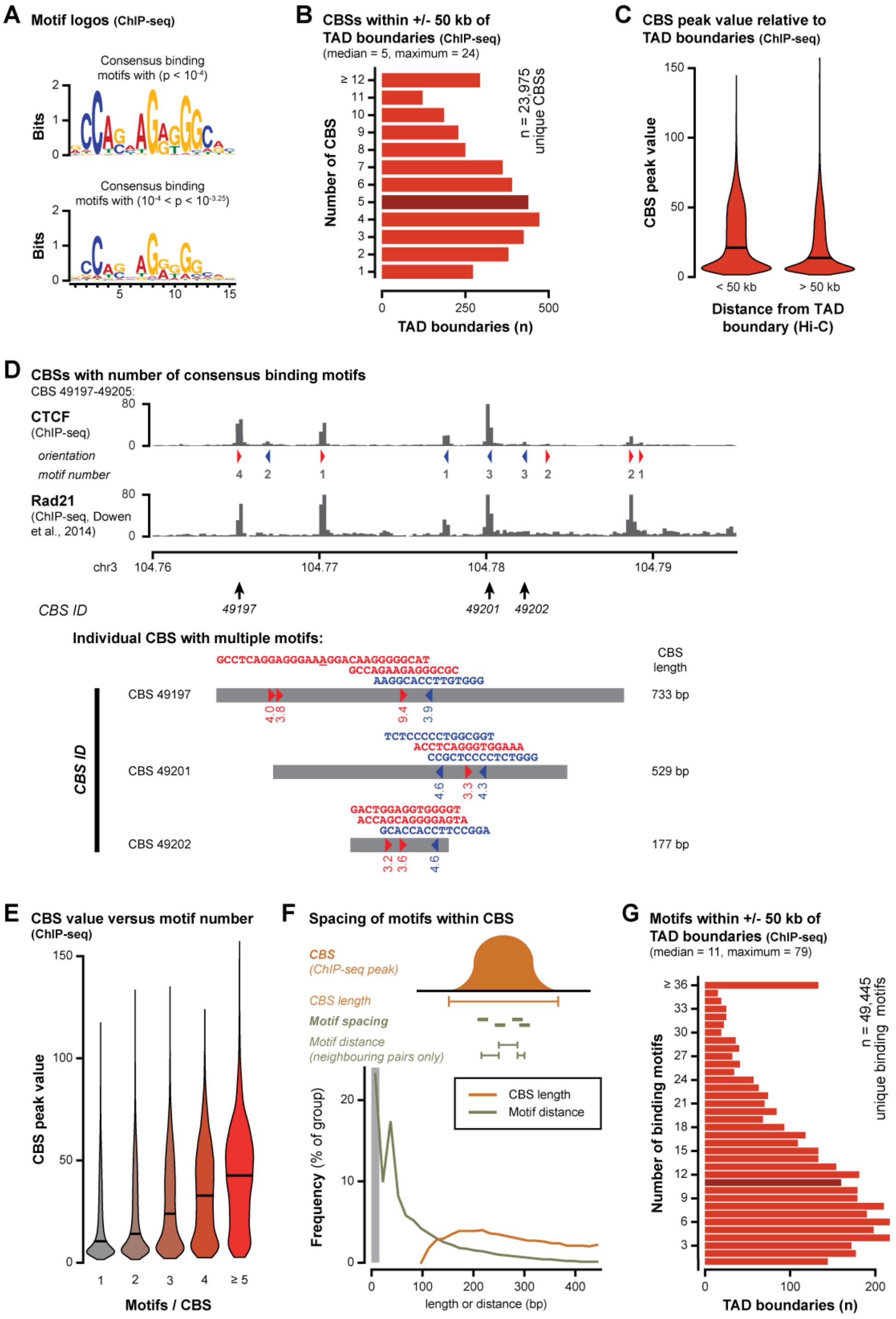
A Motif logos (ChIP-seq)
Consensus binding motifs with (p < 10-4)
Consensus binding motifs with (10-4 < p < 10-3.25)
Bits
B CBSs within +/- 50 kb of TAD boundaries (ChIP-seq)
(median = 5, maximum = 24)
Number of CBS
n = 23,975 unique CBSs
TAD boundaries (n)
C CBS peak value relative to TAD boundaries (ChIP-seq)
CBS peak value
< 50 kb
> 50 kb
Distance from TAD boundary (Hi-C)
D CBSs with number of consensus binding motifs
CBS 49197-49205:
CTCF
(ChIP-seq)
orientation
motif number
Rad21
(ChIP-seq, Dowen et al., 2014)
chr3
104.76
104.77
104.78
104.79
CBS ID
49197
49201
49202
Individual CBS with multiple motifs:
CBS length
GCCTCAGGAGGGAAAGGACAAGGGGGCAT
GCCAGAAGAGGGCGC
AAGGCACCTTGTGGG
CBS 49197
733 bp
TCTCCCCCTGGCGGT
ACCTCAGGGTGGAAA
CCGCTCCCCTCTGGG
CBS 49201
529 bp
GACTGGAGGTGGGGT
ACCAGCAGGGGAGTA
GCACCACCTTCCGGA
CBS 49202
177 bp
E CBS value versus motif number (ChIP-seq)
CBS peak value
Motifs / CBS
F Spacing of motifs within CBS
CBS (ChIP-seq peak)
CBS length
Motif spacing
Motif distance (neighbouring pairs only)
CBS length
Motif distance
Frequency (% of group)
length or distance (bp)
G Motifs within +/- 50 kb of TAD boundaries (ChIP-seq)
(median = 11, maximum = 79)
Number of binding motifs
n = 49,445 unique binding motifs
TAD boundaries (n)

Supplementary Figure 1: Multi-featured enrichment of CTCF binding at TD boundaries

A. CTCF sequence logos for highly significant (top; $p < 10^{-4}$) and lower significant (bottom; $10^{-4} < p < 10^{-3.25}$) CTCF binding motifs. P-values refer to motif significance score as determined using the MEME-suite (see Methods section). Although the lower significant logos display more variation at all positions (lower bit scores), they share similar pattern of enrichments at each position as compared to highly significant logos.

B. Significant CTCF binding sites (CBSs) in the 100 kb windows (+/- 50 kb up- and downstream) surrounding all TAD boundaries in the mESC genome. Shaded bar denotes the median value

C. CBS peak values (ChIP-seq) relative to TAD boundary distance. CBSs within 50 kb of a Hi-C boundary have, on average, a higher peak value.

D. Top: Example of a CBS cluster, consisting of 9 peaks within a 25 kb genomic interval. Six out of nine CBSs within this cluster cover multiple binding motifs. Arrowheads indicate the relative orientation of the most significant binding motif within each CBS, with the number below indicating the total number of covered binding motifs. Bottom: visualization of position and orientation of binding motifs within three CBSs. Grey bars indicate the total span of the significant ChIP-seq peak. Arrowheads indicate the position and relative orientation of motifs, with the number below indicating their significance score (-log(10) value). Red motifs have a forward orientation, blue motifs have a reverse orientation. CBS IDs are according to Supplementary Data file 1.

E. CBS peak values (ChIP-seq) relative to the number of covered binding motifs. CBSs that cover more motifs have, on average, a higher peak value.

F. Spacing of binding motifs within CBSs that contain two or more motifs. The orange line depicts the length distribution of CBSs that contain two or more binding motifs. The green line depicts the distribution of distances between all neighboring pairs of motifs within the same CBSs. Frequencies are depicted per 15 bp bin. Motif distance is calculated between the centers of neighboring motif pairs. The grey bin indicates pairs of motifs that are within 15 bp of distance and therefore are partially overlapping (making up 23% of all motif pairs). Whereas the identified CBSs can span multiple hundreds of bps, > 70% of neighboring motif pairs are within 100 bps.

G. CTCF binding motifs in the 100 kb windows (+/- 50 kb up- and downstream) surrounding all TAD boundaries in the mESC genome. Shaded bar denotes the median value.

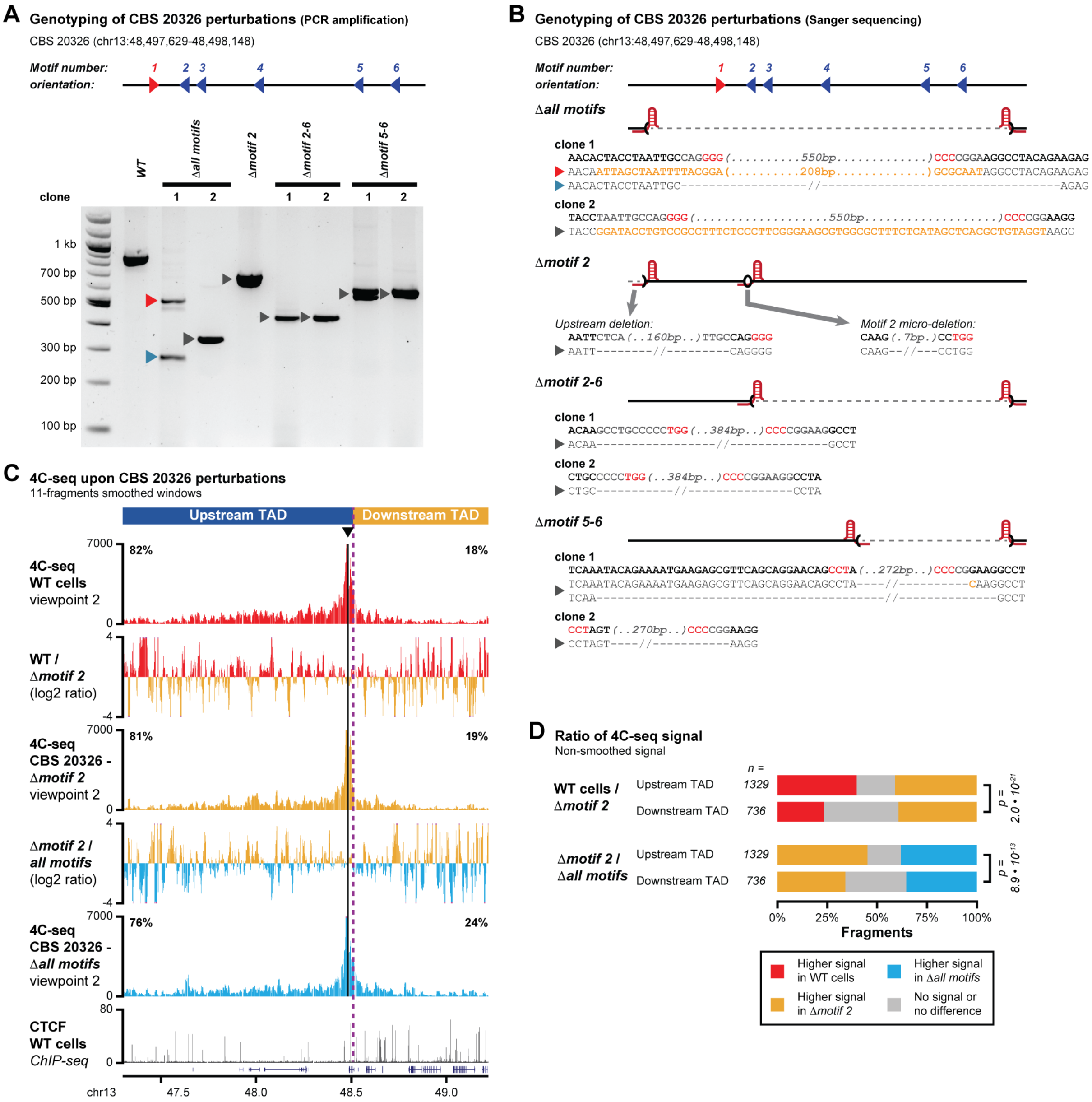
A Genotyping of CBS 20326 perturbations (PCR amplification)
CBS 20326 (chr13:48,497,629-48,498,148)
Motif number:
orientation:
WT
Δall motifs
Δmotif 2
Δmotif 2-6
Δmotif 5-6
clone
1 kb
700 bp
500 bp
300 bp
200 bp
100 bp
B Genotyping of CBS 20326 perturbations (Sanger sequencing)
CBS 20326 (chr13:48,497,629-48,498,148)
Δall motifs
clone 1
AACACTACCTAATTGCCAGGGG(..........550bp.............)CCCCGGAAGGCCTACAGAAGAG
AACAATTAGCTAATTTTACGGA(.........208bp............)GCGCAATAGGCCTACAGAAGAG
AACACTACCTAATTGC-----------------//----------------------------------AGAG
clone 2
TACCTAATTGCCAGGGG(..................550bp...................)CCCCGGAAGG
TACCGGATACCTGTCCGCCTTTCTCCCTTCGGGAAGCGTGGCGCTTTCTCATAGCTCACGCTGTAGGTAAGG
Δmotif 2
Upstream deletion:
AATTCTCA(..160bp..)TTGCCAGGGG
AATT--------//---------CAGGGG
Motif 2 micro-deletion:
CAAG(.7bp.)CCTGG
CAAG--//---CCTGG
Δmotif 2-6
clone 1
ACAAGCCTGCCCCCTGG(..384bp..)CCCCGGAAGGCCT
ACAA-----------------//--------------GCCT
clone 2
CTGCCCCCTGG(..384bp..)CCCCGGAAGGCCTA
CTGC-----------//--------------CCTA
Δmotif 5-6
clone 1
TCAAATACAGAAAATGAAGAGCGTTCAGCAGGAACAGCCTA(..272bp..)CCCCGGAAGGCCT
TCAAATACAGAAAATGAAGAGCGTTCAGCAGGAACAGCCTA----//----------CAAGGCCT
TCAA---------------------------------------//--------------GCCT
clone 2
CCTAGT(..270bp..)CCCCGGAAGG
CCTAGT----//-----------AAGG
C 4C-seq upon CBS 20326 perturbations
11-fragments smoothed windows
Upstream TAD
Downstream TAD
4C-seq WT cells viewpoint 2
82%
18%
WT / Δmotif 2 (log2 ratio)
4C-seq CBS 20326 - Δmotif 2 viewpoint 2
81%
19%
Δmotif 2 / all motifs (log2 ratio)
4C-seq CBS 20326 - Δall motifs viewpoint 2
76%
24%
CTCF WT cells ChIP-seq
chr13
47.5
48.0
48.5
49.0
D Ratio of 4C-seq signal
Non-smoothed signal
n =
WT cells / Δmotif 2
Upstream TAD 1329
Downstream TAD 736
p = 2.0 • 10^-21
Δmotif 2 / Δall motifs
Upstream TAD 1329
Downstream TAD 736
p = 8.9 • 10^-13
0%
25%
50%
75%
100%
Fragments
Higher signal in WT cells
Higher signal in Δall motifs
Higher signal in Δmotif 2
No signal or no difference

Supplementary Figure 2: Genotyping and analysis of CBS 20326 perturbations

A. Genotyping of mESC clones with deletions of one or multiple CTCF binding motifs in CBS 20326 using PCR amplification and agarose gel electrophoresis. Experiment was performed once. Above, a schematic overview of motif position within CBS 20326 is provided. Arrowheads indicate PCR bands that were further characterized by Sanger sequencing (see Supplementary Fig. 2b).

B. Genotyping of mESC clones with perturbations in CBS 20326 using Sanger sequencing. For each deletion, the CRISPR-Cas9 strategy (*i.e.* position of the gRNAs) is indicated above. For each clone, the first line indicates the WT sequence, with the PAM sequences indicated in red. Lines below indicate the consensus sequence(s) obtained from at least 8 Sanger sequencing products, obtained after cloning of the indicated PCR products (arrow heads, see Supplementary Fig. 2a). In all clones, the targeted CTCF motifs has been removed or perturbed. If only one sequence is provided, all Sanger sequencing products were identical, and we assumed that the deletion is homozygous ($p < 0.01$).

Genomic intervals between brackets [*e.g.* (..10 bp..)] indicate sequences of the indicated size that are not visualized. Intervals indicated by dashes (*e.g.* ---//---) indicate sequences that are deleted. Intervals indicated in orange represent sequences that are integrated or otherwise repositioned. Similar to a recent report[1], we detect a number of exogenous integrations:

- *Δall motifs* – clone 1: integration of a 233 bp exogenous DNA sequence from the *E. coli* genome.
- *Δall motifs* – clone 2: integration of a 64 bp exogenous DNA sequence from the plasmid used to express the Cas9 protein and gRNAs[2].

The *Δmotif 2* clone is the unforeseen outcome of our strategy to create a *Δmotif* 1-2 deletion, which generated a homozygous dual deletion consisting of a 160 bp deletion downstream of the left gRNA and a 7 bp deletion at the right gRNA. The micro-deletion caused by the right gRNA perturbs CTCF binding motif 2.

C. 4C-seq tracks within the 2 TADs surrounding a TAD boundary on chromosome 13 in mESCs. Top track (red) shows data from WT cells, middle track (orange) shows data from CBS 20326 Δ*motif 2* cells and bottom track (blue) shows data from CBS 20326 Δ*all motifs* cells. In-between the tracks, a log(2) track is provided.
The purple line indicates the TAD boundary of interest. The black arrowhead and line indicate the position of the 4C-seq viewpoint. The viewpoint is located 15 kb upstream from CBS 20326. Percentages indicate the fraction of contacts upstream or downstream of the boundary. The removal of increasingly more CTCF binding motifs causes a mild increase in contacts downstream of the boundary.

D. Pairwise analysis of 4C-seq ratios within the 2 TADs surrounding the TAD boundary upon perturbations of CBS 20326. In this approach, the contribution of the bins with high signal (*i.e.* those that directly surround the viewpoint and the TAD boundary) is dampened, thereby improving the identification of changes within the entire domain. In the TAD where the viewpoint is located (upstream), the number of bins with positive and negative signals is comparable between conditions. In the downstream TAD, the number of bins with positive signal is strongly increased when more motifs within CBS 20326 have been removed. Significance: G-test of independence.

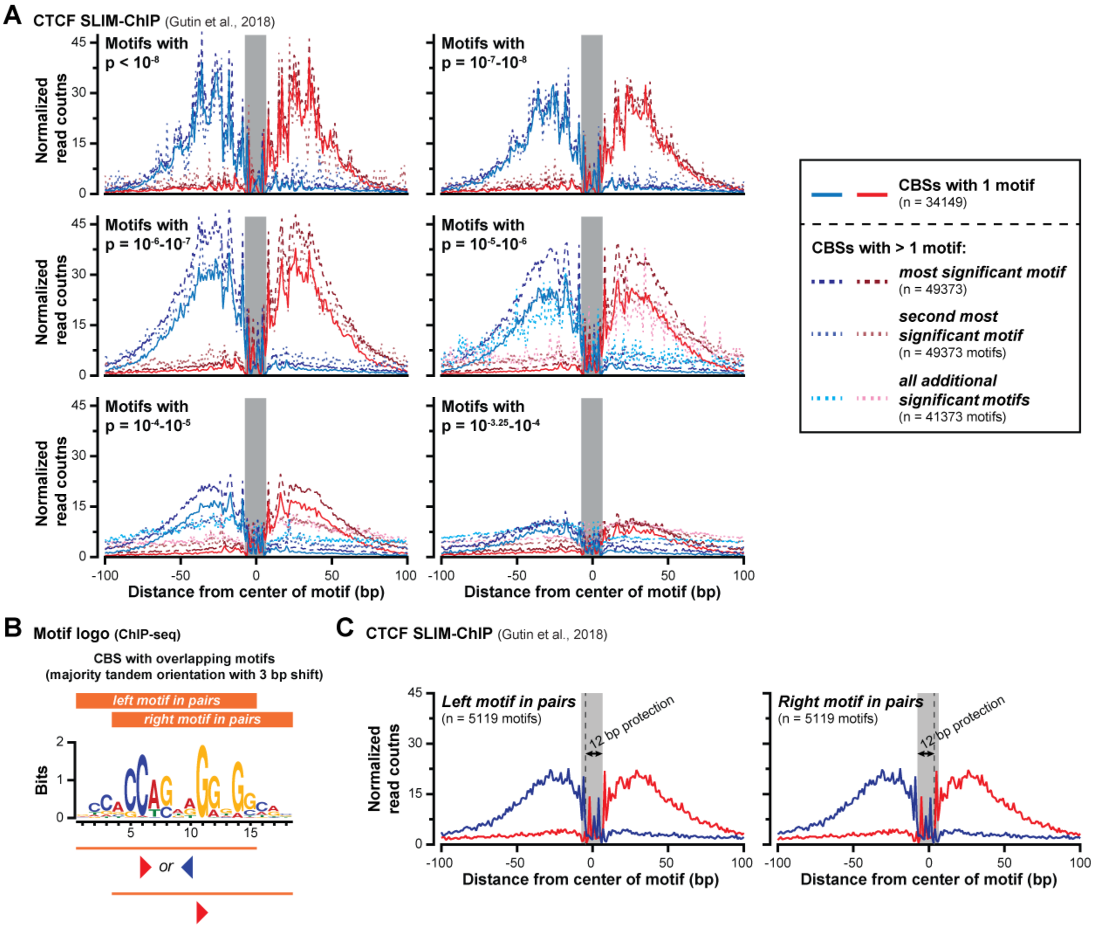

Supplementary Figure 3: SLIM-ChIP analysis of CTCF binding

A. Normalized pile-up of SLIM-ChIP[3] read start positions for motifs sorted on their significance (different graphs, with p-value range indicated in the graph) and their presence in different types of CBSs (color coding of lines, see legend). The grey shaded area overlaps the 15 bp CTCF binding motif. Categories with fewer than 100 motifs have been excluded. P-values refer to motif significance score as determined using the MEME-suite (see Methods section). See also Fig. 2c and Supplemental Data file 2.

B. Combined 18 bp sequence logo for CTCF binding motif pairs that are overlapping with a 3 bp shift. In about 75% of these overlapping pairs, the motifs are in tandem (++), whereas in about 25% of pairs the motifs are in a divergent orientation (-+). The convergent orientation (+-) is never detected.

C. Normalized pile-up of SLIM-ChIP read start positions for CTCF binding motif pairs that are overlapping in tandem with a 3 bp shift. Left: pile-up of the left motif in the overlapping pairs. Right: pile-up of the right motif in the overlapping pairs. The grey box indicates the 15 bp motif that is protected in normal binding motifs and the dashed line demarcates the 3 bp that are not protected in the overlapping pairs.

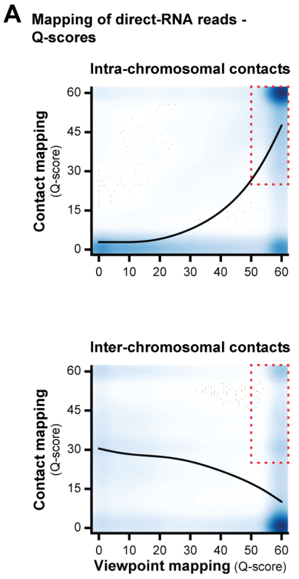

B **Comparision to other multi-contact assays**
(all data analyzed using Nano-C data analysis pipeline)

	# unique reads (viewpoint containing, genome-wide contacts)	# unique multi-contact reads (viewpoint containing, genome-wide contacts)	Multiplex index (= number of viewpoints/experiment)	Platform	# replicates	Reference
**Nano-C** (WT runs, 13 viewpoints)	65,701	25,599	10-15	MinION - direct RNA (Oxford Nanopore Technologies)	10*	This study
**MC-4C** (*Hbb-b1* viewpoint)	1,131,725	1,023,861	1	MinION - DNA (Oxford Nanopore Technologies)	5	Allahyar et al., 2018
**Tri-C** (*R2, HS-39, HS2* and *3'HS1* viewpoints)	151,164,972	55,857,804	4	Next-seq 500 - PE 150 bp (Illumina)	14	Oudelaar et al., 2018

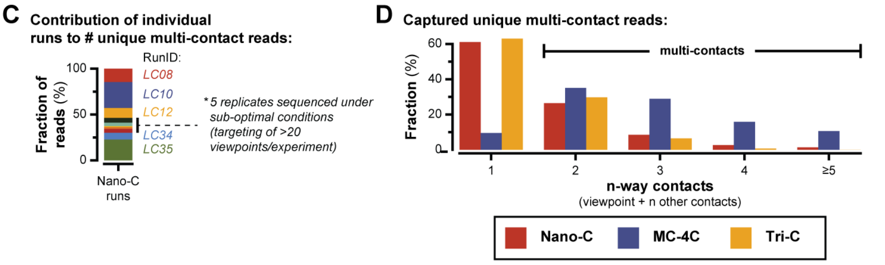

Supplementary Figure 4: Nano-C read filtering and comparison to other multi-contact assays

A. Q-score for reads that map both to the viewpoint (horizontal axis; determined in the second step of our analysis) and to other sites in the genome (vertical axis; determined in the third step of our analysis). Top graph: mapping scores for contact pairs where the viewpoint and the identified contact are on the same chromosome (intra-chromosomal contact). Bottom graph: mapping scores for contact pairs where the viewpoint and the identified contact are on different chromosomes (inter-chromosomal contact). Most intra-chromosomal contact pairs have high Q-scores for both the viewpoint and the identified contact (upper-right corner), but a smaller population has a low mapping Q-score for the identified contact (bottom-right corner). Most inter-chromosomal contact pairs have high Q-scores for the viewpoint and a low Q-score for the identified contact. A smaller population is present with high Q-score for both the viewpoint and the contact. Considering that contacts were mapped to the entire genome, we expect similar Q-scores for mapping anywhere in the genome. As high mapping Q-scores were enriched on the same chromosome, we assume that most low Q-scores for inter-chromosomal mappings represent low-quality non-specific mapping. For this reason, we only retain reads with high Q-scores for both the viewpoint and the contact (red box).

B. Comparison of key numbers for Nano-C and published Multi-Contact 4C (MC-4C)[4] and Tri-C[5] assays. Data was compared for reads mapping to the indicated viewpoints using our Nano-C analysis pipeline (with minor adjustments if required for data from the other assays). Although Nano-C generates fewer unique reads, it requires considerably fewer and less costly sequencing experiments to obtain data for 10-15 viewpoints (see Multiplex index). See also Supplementary Fig. 4c and Supplementary Data file 3.

C. The majority of WT Nano-C data was obtained in 5 runs out of 10 total, due to suboptimal conditions for the 5 other runs (inclusion of too many viewpoints). See Material and Methods section and Supplementary Data file 3 for detail.

D. Percentages of unique pair-wise (1-way) and multi-way (≥ 2-way) reads captured by Nano-C, MC-4C and Tri-C.

Supplementary Figure 5: Comparison between Nano-C, Hi-C and 4C-seq

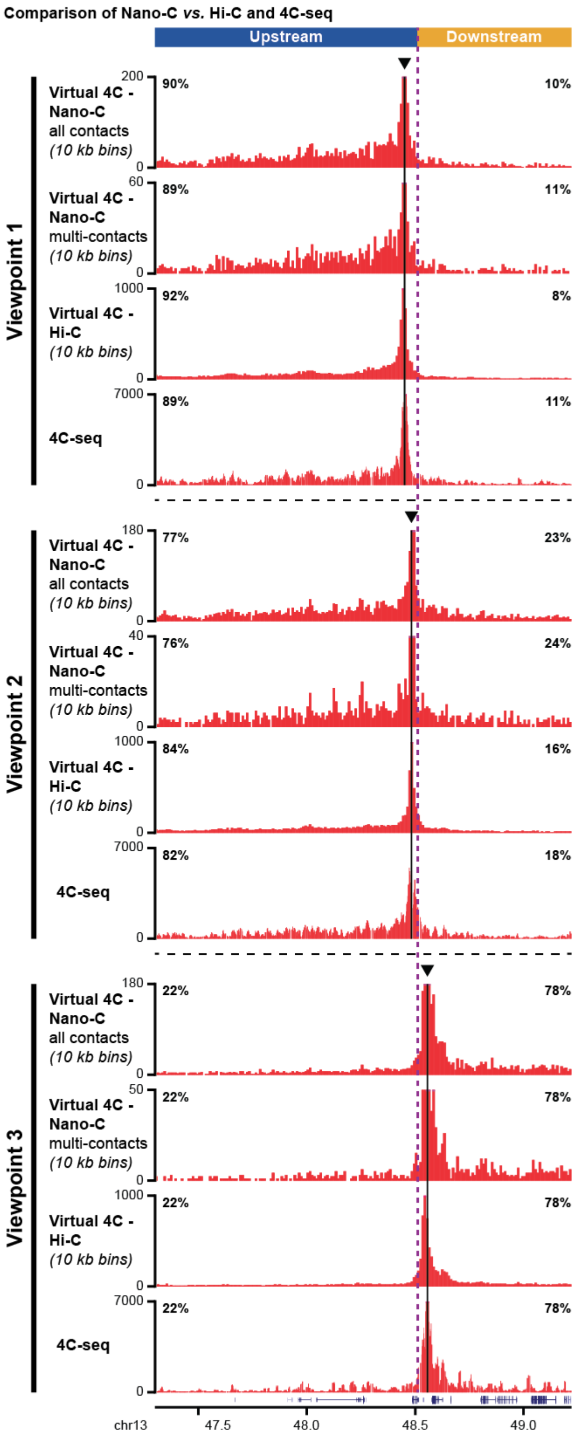

Nano-C, Hi-C and 4C-seq tracks are compared within the 2 TADs surrounding a TAD boundary on chromosome 13 in WT mESCs. For each of the bins containing a Nano-C viewpoint, virtual 4C tracks were generated for Nano-C and Hi-C data at 10 kb resolution.

For each viewpoint, track 1 shows a virtual 4C track from pairwise Nano-C contacts. Track 2 shows a virtual 4C track from Nano-C multi-contacts. Track 3 shows a virtual 4C track from Hi-C data. Track 4 shows newly-generated 4C-seq data (viewpoint overlapping the Nano-C viewpoints, patterns smoothed using an 11 NlaIII fragments running-mean).

The dashed purple line indicates the position of the TAD boundary of interest. The black arrowheads and lines indicate the position of the viewpoints. Percentages indicate the fraction of contacts upstream or downstream of the boundary.

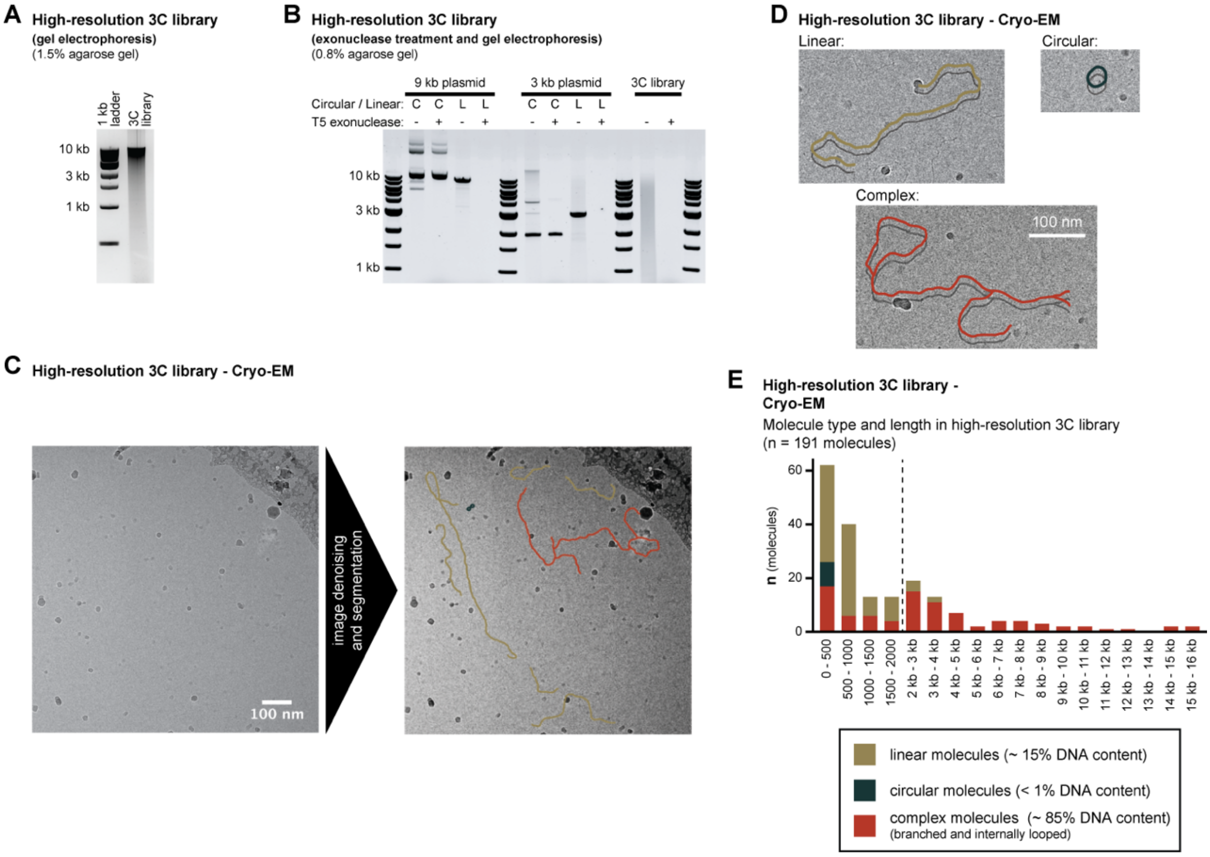

Supplementary Figure 6: Cryo-EM visualization of 3C libraries

A. Electrophoresis of a high-resolution 3C library on a 1.5% agarose gel (NlaIII digestion), showing a discrete high molecular weight band running at around 10 kb. Experiment was performed once.

B. T5 exonuclease treatment to assess the nature of high-resolution 3C libraries. T5 exonuclease treatment degrades DNA molecules that have open ends (*i.e.* non-circular DNA), whereas circular molecules remain intact. The presence or absence of degradation was analyzed by electrophoresis on a 0.8% agarose gel of indicated DNA molecules, including two control plasmids with and without linearization. Experiments were performed once.
3C libraries are digested by T5 exonuclease, thereby confirming that the majority of molecules are not circular (see also ref. 6). We noted nonetheless that the size distribution of the undigested 3C library on a 0.8% agarose gel is different from a 1.5% agarose gel (Supplementary Fig. 6a), suggesting that 3C molecules are of an unconventional nature.

C.,D. Visualization of individual DNA molecules in a high-resolution 3C library by Cryo-EM (cryo-electron microscopy). DNA molecules were trapped within a thin vitreous ice layer and imaged at cryo-temperature. Three types of topologies were detected: linear molecules (highlighted in brown), circular molecules (black) and molecules with complex topologies (branched and internally looped; red).

E. Quantitation of 3C fragments by Cryo-EM. The length of the DNA molecules was converted to base-pairs from the measured length. Among the 191 molecules analyzed, the DNA molecules larger than 4 kb consisted only of complex topologies, while linear and circular molecules were mostly observed for fragments < 2 kb.

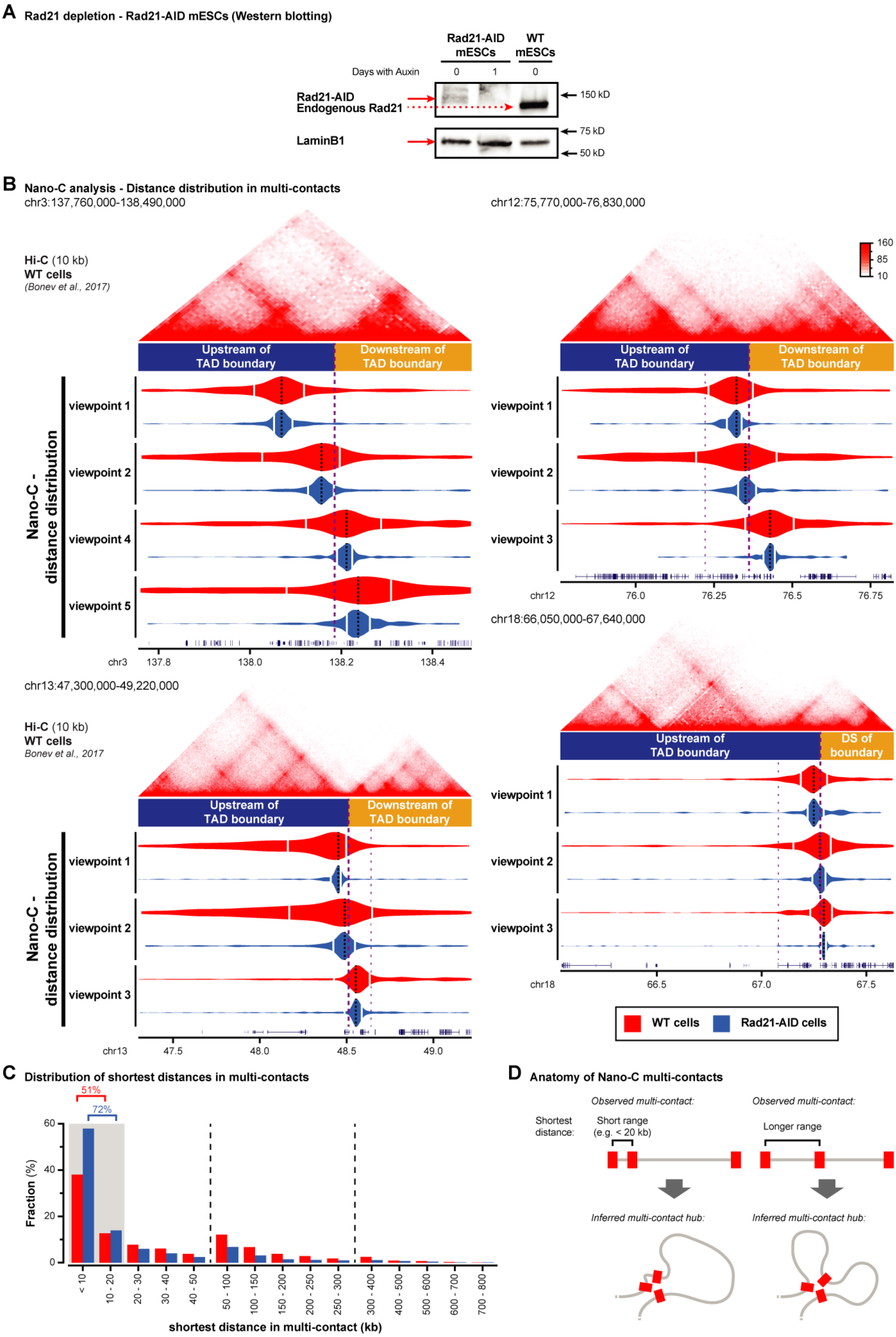

A Rad21 depletion - Rad21-AID mESCs (Western blotting)
Rad21-AID mESCs
WT mESCs
Days with Auxin
0
1
0
Rad21-AID
Endogenous Rad21
LaminB1
150 kD
75 kD
50 kD
B Nano-C analysis - Distance distribution in multi-contacts
chr3:137,760,000-138,490,000
chr12:75,770,000-76,830,000
Hi-C (10 kb)
WT cells
(Bonev et al., 2017)
160
85
10
Upstream of TAD boundary
Downstream of TAD boundary
Nano-C - distance distribution
viewpoint 1
viewpoint 2
viewpoint 4
viewpoint 5
viewpoint 3
chr3
137.8
138.0
138.2
138.4
chr12
76.0
76.25
76.5
76.75
chr18:66,050,000-67,640,000
chr13:47,300,000-49,220,000
Bonev et al., 2017
DS of boundary
chr13
47.5
48.0
48.5
49.0
chr18
66.5
67.0
67.5
WT cells
Rad21-AID cells
C Distribution of shortest distances in multi-contacts
51%
72%
Fraction (%)
60
40
20
0
< 10
10 - 20
20 - 30
30 - 40
40 - 50
50 - 100
100 - 150
150 - 200
200 - 250
250 - 300
300 - 400
400 - 500
500 - 600
600 - 700
700 - 800
shortest distance in multi-contact (kb)
D Anatomy of Nano-C multi-contacts
Observed multi-contact:
Shortest distance:
Short range
(e.g. < 20 kb)
Longer range
Inferred multi-contact hub:

Supplementary Figure 7: Nano-C analysis of multi-contacts at TAD boundaries in WT and Rad21-AID mESCs

A. Validation of Rad21 degradation upon Auxin-treatment in Rad21-AID mESCs[7]. The solid arrow indicates the Rad21-AID fusion protein. The dashed arrow indicates the non-tagged Rad21 protein in WT cells. Experiment was performed once. Original blots provided in Supplementary Fig. 13.

B. Distance distribution of Nano-C multi-contacts for 13 viewpoints in the two surrounding TADs that border four boundaries in WT cells and Rad21-AID cells. Violin plots indicate distance distribution of up- and downstream interactions of the viewpoint, with white lines indicating the median distances. Reanalyzed Hi-C data[8] from WT cells is indicated above. Thick purple lines indicate TAD boundaries of interest and smaller lines indicate nearby other boundaries. All viewpoints are located close to the TAD boundaries of interest. Distances in Rad21-AID cells are strongly reduced and distributed more symmetrical as compared to WT cells.

C. Binned distribution of shortest distances within Nano-C multi-contacts in WT and Rad21-AID cells. The grey shaded area indicates the fraction of multi-contacts with shortest distances < 20 kb, which represent > 50% of multi-contacts (see indicated cumulative fraction in the first two bins).

D. Cartoon showing the anatomy of Nano-C multi-contacts. The majority of multi-contacts are made up of 2-way contacts (viewpoints + 2 other contacts, see Supplementary Fig. 4d). We speculate that multi-contacts that contain a pair of nearby fragments (< 20 kb; "short range") are the representation of single DNA loops, whereas multi-contacts that contain more distant interactions only (> 20 kb; "longer range") represent higher-order configurations consisting of two or more loops.

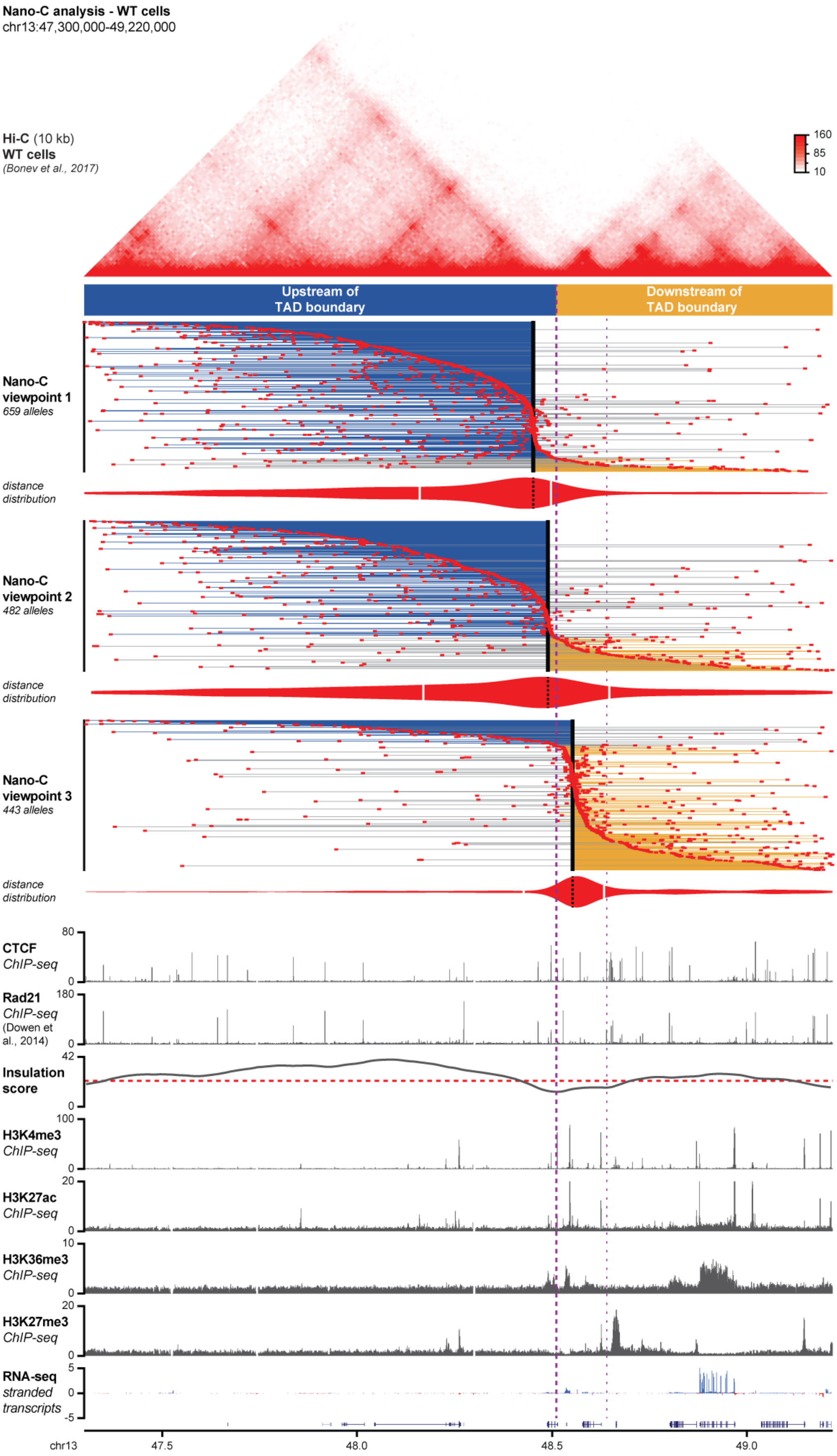
Nano-C analysis - WT cells
chr13:47,300,000-49,220,000
Hi-C (10 kb)
WT cells
(Bonev et al., 2017)
160
85
10
Upstream of TAD boundary
Downstream of TAD boundary
Nano-C viewpoint 1
659 alleles
distance distribution
Nano-C viewpoint 2
482 alleles
distance distribution
Nano-C viewpoint 3
443 alleles
distance distribution
CTCF ChIP-seq
80
0
Rad21 ChIP-seq (Dowen et al., 2014)
180
0
Insulation score
42
0
H3K4me3 ChIP-seq
100
0
H3K27ac ChIP-seq
20
0
H3K36me3 ChIP-seq
10
0
H3K27me3 ChIP-seq
20
0
RNA-seq stranded transcripts
5
0
-5
chr13
47.5
48.0
48.5
49.0

Supplementary Figure 8: Nano-C analysis of multi-contacts at a TAD boundary in WT mESCs

Nano-C multi-contacts for three viewpoints in the two surrounding TADs that are located close to a boundary on chromosome 13 in WT cells. Individual lines in the plots represent single multi-contact reads, with the viewpoint depicted as a black box and each contact as a red box. The color of the lines indicates if all interactions are in the TAD upstream of the boundary (blue lines), the TAD downstream of the boundary (orange lines) or in both TADs (grey lines). Multi-contacts are sorted on the contact that is nearest to the viewpoint. Violin plots indicate distance distribution of up- and downstream interactions of the viewpoint, with white lines indicating the median distances. Reanalyzed Hi-C data[8] is indicated above. ChIP-seq tracks for CTCF, Rad21[9], H3K4me3, H3K27ac, H3K36me3 and H3K27me3, strand-specific RNA-seq tracks and the Hi-C insulation score (red line: cut-off) are depicted below. The thick purple line indicates the TAD boundary of interest and the smaller line indicates a nearby boundary. Notice that the TAD upstream of the boundary is mostly transcriptionally inactive, with only a bivalently marked promoter being present (H3K4me3 and H3K27me3). The TAD downstream of the boundary contains several actively transcribed promoters.

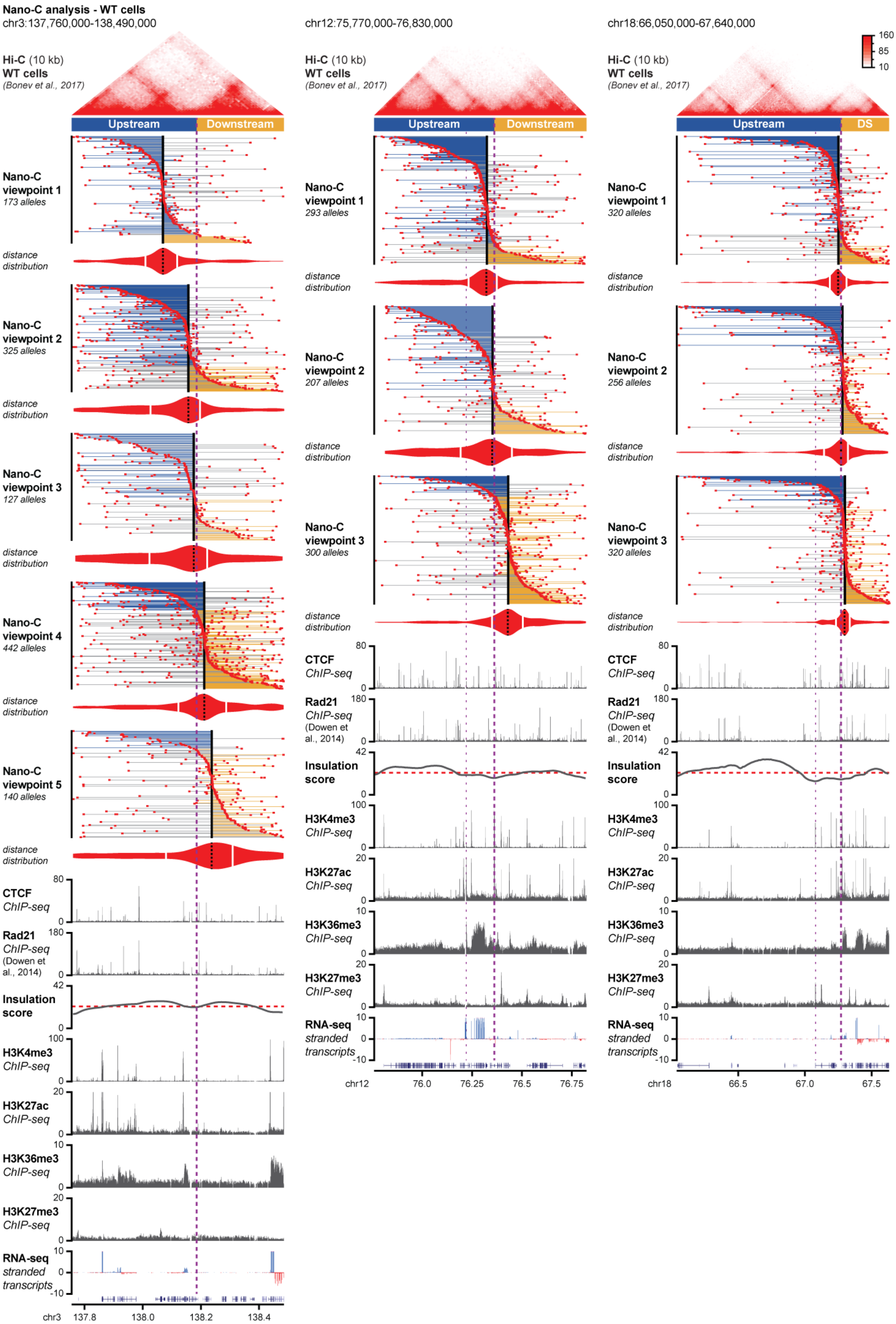

Nano-C analysis - WT cells
chr3:137,760,000-138,490,000
chr12:75,770,000-76,830,000
chr18:66,050,000-67,640,000
Hi-C (10 kb)
WT cells
(Bonev et al., 2017)
160
85
10
Upstream
Downstream
DS
Nano-C viewpoint 1
173 alleles
293 alleles
320 alleles
distance distribution
Nano-C viewpoint 2
325 alleles
207 alleles
256 alleles
Nano-C viewpoint 3
127 alleles
300 alleles
320 alleles
Nano-C viewpoint 4
442 alleles
Nano-C viewpoint 5
140 alleles
CTCF ChIP-seq
Rad21 ChIP-seq (Dowen et al., 2014)
Insulation score
H3K4me3 ChIP-seq
H3K27ac ChIP-seq
H3K36me3 ChIP-seq
H3K27me3 ChIP-seq
RNA-seq stranded transcripts
chr3
137.8
138.0
138.2
138.4
chr12
76.0
76.25
76.5
76.75
chr18
66.5
67.0
67.5

Supplementary Figure 9: Nano-C analysis of multi-contacts at three TAD boundaries in WT mESCs

Nano-C multi-contacts for viewpoints are located close to three different boundaries in WT cells. Multi-contacts are shown for the two surrounding TADs. Individual lines in the plots represent single multi-contact reads, with the viewpoint depicted as a black box and each contact as a red box. The color of the lines indicates if all interactions are in the TAD upstream of the boundary (blue lines), the TAD downstream of the boundary (orange lines) or in both TADs (grey lines). Multi-contacts are sorted on the contact that is nearest to the viewpoint. Violin plots indicate distance distribution of up- and downstream interactions of the viewpoint, with white lines indicating the median distances. Reanalyzed Hi-C data[8] is indicated above. ChIP-seq tracks for CTCF, Rad21[9], H3K4me3, H3K27ac, H3K36me3 and H3K27me3, strand-specific RNA-seq tracks and the Hi-C insulation score (red line: cut-off) are depicted below. Thick purple lines indicate the TAD boundary of interest and smaller lines indicate nearby boundaries.

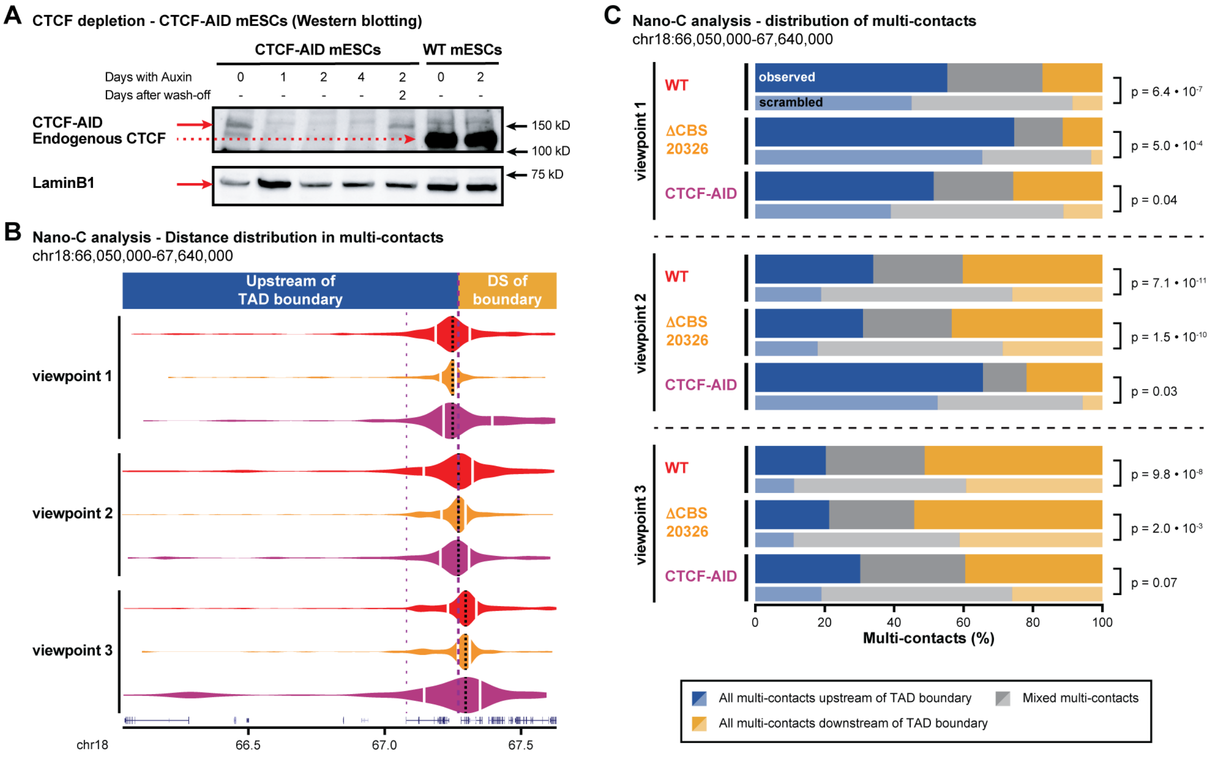

Supplementary Figure 10: Nano-C analysis of multi-contacts in WT, ΔCBS 20326 and CTCF-AID mESCs

A. Validation of CTCF degradation upon Auxin-treatment in CTCF-AID mESCs[10]. The solid arrow indicates the CTCF-AID fusion protein. The dashed arrow indicates the non-tagged CTCF protein in WT cells. Experiment was performed once. Original blots provided in Supplementary Fig. 13.

B. Distance distribution of Nano-C multi-contacts for three viewpoints in the two surrounding TADs that border a boundary on chromosome 18 in WT, ΔCBS 20326 and CTCF-AID cells. Violin plots indicate distance distribution of up- and downstream interactions of the viewpoint, with white lines indicating the median distances. The thick purple line indicates the TAD boundary of interest and the smaller line indicates a nearby boundary. All viewpoints are located close to the TAD boundaries of interest. Distances of interactions that cross the TAD boundary in CTCF-AID cells are considerably increased as compared to WT and ΔCBS 20326 (a CBS on a different chromosome) cells.

C. Distribution of Nano-C multi-contacts in the two surrounding TADs that border a boundary on chromosome 18 for three viewpoints in WT, ΔCBS 20326 and CTCF-AID cells. Scrambled distributions of multi-contacts were obtained after randomly assigning contacts up- and downstream into multi-contacts. Significance: G-test of independence.

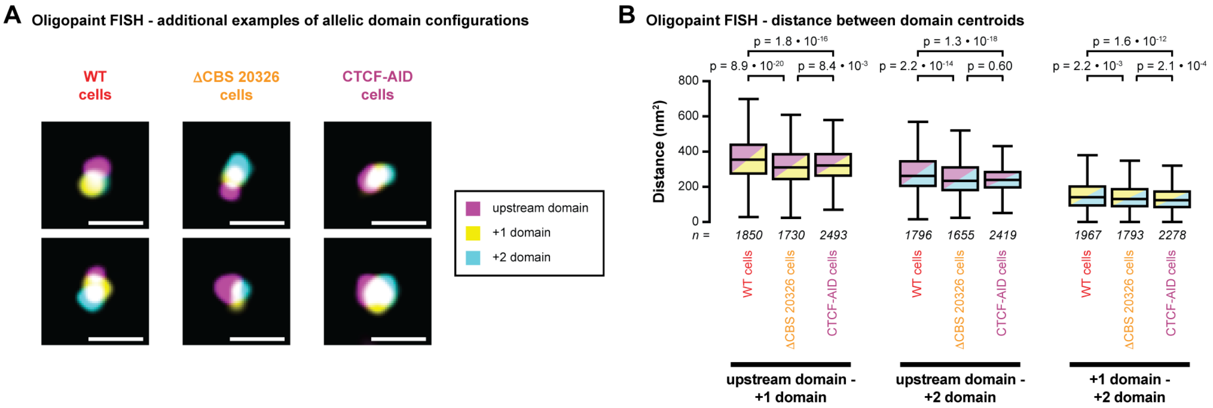

Supplementary Figure 11: Oligopaint DNA-FISH in WT, ΔCBS 20326 and CTCF-AID mESCs

A. Additional representative FISH images for the three cell lines. Scale bar = 1 µm (see Fig. 6f).

B. Distances between centroids of the indicated domains in the three cell lines. Boxplots were generated in R using standard settings, where the box represents the range between the first and third quartile values and the center line the median value. Whiskers represent the first or third quartile +/- the interquartile range. The number of analyzed alleles is indicated below. Shading refers to the pairs of domains that are analyzed. Significance: two-tailed Mann-Whitney test.

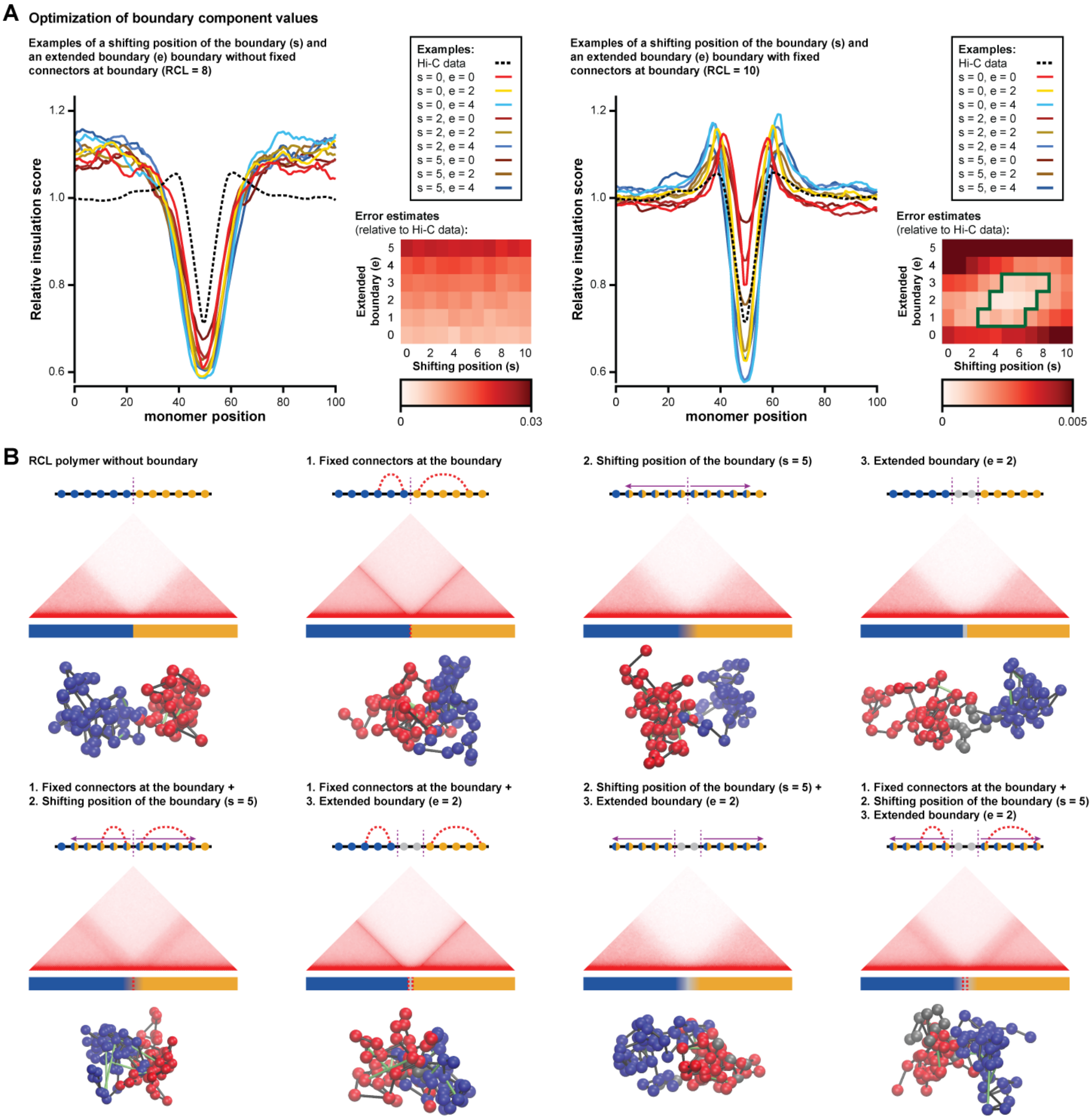
A
Optimization of boundary component values
Examples of a shifting position of the boundary (s) and an extended boundary (e) boundary without fixed connectors at boundary (RCL = 8)
Examples:
Hi-C data
s = 0, e = 0
s = 0, e = 2
s = 0, e = 4
s = 2, e = 0
s = 2, e = 2
s = 2, e = 4
s = 5, e = 0
s = 5, e = 2
s = 5, e = 4
Relative insulation score
monomer position
Error estimates (relative to Hi-C data):
Extended boundary (e)
Shifting position (s)
Examples of a shifting position of the boundary (s) and an extended boundary (e) boundary with fixed connectors at boundary (RCL = 10)
B
RCL polymer without boundary
1. Fixed connectors at the boundary
2. Shifting position of the boundary (s = 5)
3. Extended boundary (e = 2)
1. Fixed connectors at the boundary + 2. Shifting position of the boundary (s = 5)
1. Fixed connectors at the boundary + 3. Extended boundary (e = 2)
2. Shifting position of the boundary (s = 5) + 3. Extended boundary (e = 2)
1. Fixed connectors at the boundary + 2. Shifting position of the boundary (s = 5) + 3. Extended boundary (e = 2)

Supplementary Figure 12: Optimization of a modified Randomly Cross-Linked (RCL) polymer model to reproduce TAD boundary characteristics

A. Relative insulation score for a modified Randomly Cross-Linked (RCL) polymer model with varying parameters for shifting positions (s) and the size of the extended boundary (e). Two sequential TADs are concatenated into two blocks of RCL polymers, each of them composed of 100 monomers linearly connected by harmonic springs with an addition of a fixed number (N_c) of random connectors between non-sequential monomers. All parameters for the RCL polymer model are as reported previously[11], unless mentioned otherwise. All graphs and error estimates are based on the 50 monomers that are located up- and downstream of the boundary (indicated as monomers 1-100 in the figures, with the (average) position of the boundary between monomers 50 and 51).

Left: models without fixed connectors at the boundary; right: models with fixed connectors at the boundary. The black dashed line represents the average relative insulation score at all boundaries in WT mESCs, as determined from reanalyzed Hi-C data[8]. On the right of each graph, matrices with error estimates for the insulation score relative to Hi-C data. Notice different color gradients for the both matrices. In the absence of fixed connectors at the boundary, error values are smallest when using N_c = 8 random connectors. In this setup, the s-value has no consistent impact and increasing the e-value increases the error estimate. In the presence of fixed connectors at the boundary, error values are smallest when using N_c = 10 random connectors. In this setup, an optimal zone of error values is obtained around s = 5 and e = 2 (green box).

B. Visualizations of the eight scenarios that incorporate fixed connectors at the boundary, a shifting position of the boundary (s = 5) and an extended boundary (e = 2) into the RCL polymer model. Top: linear depiction of the 12 monomers surrounding the boundary, with blue monomers belonging to TAD 1, orange monomers to TAD 2, blue/orange monomers to either TAD due to the shifting position of the boundary and grey monomers to the gap without random connectors. Middle: *in-silico* Hi-C map of the 100 monomers that surround the (average) boundary. Bottom: examples of individual polymer realizations. *in-silico* Hi-C maps were computed by averaging 10^3 polymer realizations.

Uncropped and unprocessed scans of Western blots for Rad21 and LaminB (Supplemental Fig. 7A)

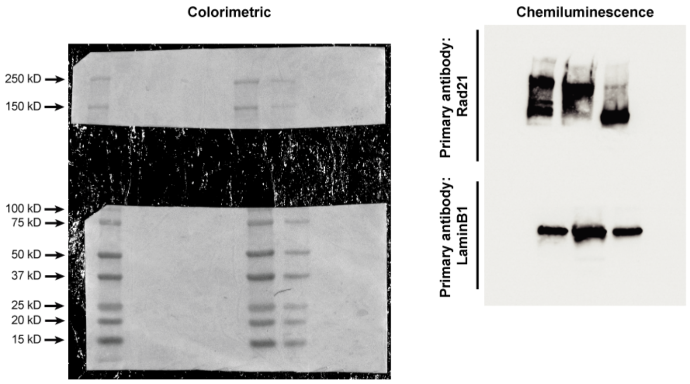

Uncropped and unprocessed scans of Western blots for CTCF and LaminB (Supplemental Fig. 10A)

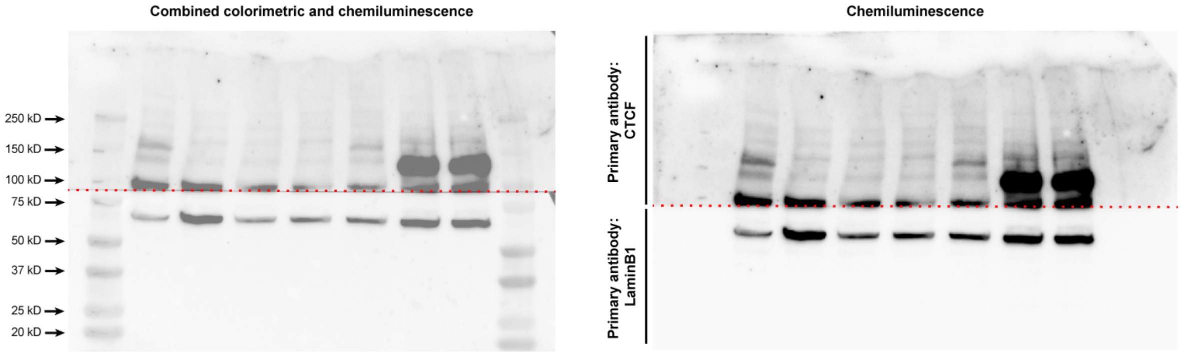

After transfer, membrane was cut to permit separate incubation with CTCF (top part) and LaminB (bottom part) antibody. Colorimetric and chemilunescent imaging was performed simultaneously.

Supplementary Figure 13: Uncropped and unprocessed scans of Western blots

Supplementary Data files

Supplementary Data file 1. CTCF peaks and motifs. *(.xlsx)*

List of identified CTCF peaks in WT mESCs and the CTCF motifs contained within.

Supplementary Data file 2. SLIM-ChIP motif numbers in CBS categories. *(.xlsx)*

List of CTCF motif numbers in each category of CBSs in WT mESCs, used for the filtering of SLIM-ChIP data[3].

Supplementary Data file 3. Overview of Nano-C runs. *(.xlsx)*

List of Nano-C runs, including cell types, viewpoints and number of contacts per viewpoint.

Supplementary Data file 4. gRNA sequences, primers and probes. *(.xlsx)*

List of gRNAs used for genome editing and *in-vitro* CRISPR-Cas9 cutting (ELF-Clamp), primers used for RT-qPCR, genotyping and 4C-seq, and biotinylated probes used for site-specific T7 promoter fusion (ELF-Clamp).

Supplementary Data file 5. TADs. *(.bed)*

BED file with start and end coordinates of TADs in WT mESCs that were identified from reanalyzed Hi-C data[8].

Supplementary Data file 6. Insulation scores. *(.bedgraph)*

BedGraph file with insulation scores for all 10kb bins in the WT mESC genome, as determined from reanalyzed Hi-C data[8].

Supplementary references

1 Geng, K. *et al.* CRISPR/Cas9 deletions induce adverse on-target genomic effects leading to functional DNA in human cells. *bioRxiv*, 2021.2007.2001.450727, doi:10.1101/2021.07.01.450727 (2021).

2 Ran, F. A. *et al.* Genome engineering using the CRISPR-Cas9 system. *Nat Protoc* **8**, 2281-2308, doi:10.1038/nprot.2013.143 (2013).

3 Gutin, J. *et al.* Fine-Resolution Mapping of TF Binding and Chromatin Interactions. *Cell Rep* **22**, 2797-2807, doi:10.1016/j.celrep.2018.02.052 (2018).

4 Allahyar, A. *et al.* Enhancer hubs and loop collisions identified from single-allele topologies. *Nat Genet* **50**, 1151-1160, doi:10.1038/s41588-018-0161-5 (2018).

5 Oudelaar, A. M. *et al.* Single-allele chromatin interactions identify regulatory hubs in dynamic compartmentalized domains. *Nat Genet* **50**, 1744-1751, doi:10.1038/s41588-018-0253-2 (2018).

6 Tavares-Cadete, F., Norouzi, D., Dekker, B., Liu, Y. & Dekker, J. Multi-contact 3C reveals that the human genome during interphase is largely not entangled. *Nat Struct Mol Biol* **27**, 1105-1114, doi:10.1038/s41594-020-0506-5 (2020).

7 Liu, N. Q. *et al.* WAPL maintains a cohesin loading cycle to preserve cell-type-specific distal gene regulation. *Nat Genet* **53**, 100-109, doi:10.1038/s41588-020-00744-4 (2021).

8 Bonev, B. *et al.* Multiscale 3D Genome Rewiring during Mouse Neural Development. *Cell* **171**, 557-572 e524, doi:10.1016/j.cell.2017.09.043 (2017).

9 Dowen, J. M. *et al.* Multiple structural maintenance of chromosome complexes at transcriptional regulatory elements. *Stem Cell Reports* **1**, 371-378, doi:10.1016/j.stemcr.2013.09.002 (2013).

10 Nora, E. P. *et al.* Targeted Degradation of CTCF Decouples Local Insulation of Chromosome Domains from Genomic Compartmentalization. *Cell* **169**, 930-944 e922, doi:10.1016/j.cell.2017.05.004 (2017).

11 Shukron, O. & Holcman, D. Transient chromatin properties revealed by polymer models and stochastic simulations constructed from Chromosomal Capture data. *PLoS computational biology* **13**, e1005469, doi:10.1371/journal.pcbi.1005469 (2017).
