# Supplementary material for: Multi-feature clustering of CTCF binding creates robustness for loop extrusion blocking and Topologically Associating Domain boundaries
Source: Nat Commun. 2023 Sep 12;14:5615. doi: 10.1038/s41467-023-41265-y (PMC10497529; doi:10.1038/s41467-023-41265-y)
Supplement: Supplementary file 3 — Description of Additional Supplementary Files [file 41467_2023_41265_MOESM3_ESM.pdf]

Description of Additional Supplementary Files

File Name: Supplementary Data 1
Description: List of identified CTCF peaks in WT mESCs and the CTCF motifs contained within.

File Name: Supplementary Data 2
Description: List of CTCF motif numbers in each category of CBSs in WT mESCs, used for the filtering of SLIM-ChIP data.

File Name: Supplementary Data 3
Description: List of Nano-C runs, including cell types, viewpoints and number of contacts per viewpoint.

File Name: Supplementary Data 4
Description: List of gRNAs used for genome editing and *in-vitro* CRISPR-Cas9 cutting (ELFClamp), primers used for RT-qPCR, genotyping and 4C-seq, and biotinylated probes used for site-specific T7 promoter fusion (ELF-Clamp).

File Name: Supplementary Data 5
Description: BED file with start and end coordinates of TADs in WT mESCs that were identified from reanalyzed Hi-C data.

File Name: Supplementary Data 6
Description: BedGraph fiule with insulation scores for all 10kb bins in the WT mESC genome, as determined from reanalyzed Hi-C data.
