# Supplementary material for: Multi-feature clustering of CTCF binding creates robustness for loop extrusion blocking and Topologically Associating Domain boundaries
Source: Nat Commun. 2023 Sep 12;14:5615. doi: 10.1038/s41467-023-41265-y (PMC10497529; doi:10.1038/s41467-023-41265-y)
Supplement: Supplementary file 10 — Reporting Summary [file 41467_2023_41265_MOESM10_ESM.pdf]

nature portfolio

Corresponding author(s): Daan NOORDERMEER

Last updated by author(s): Aug 10, 2023

Reporting Summary

Nature Portfolio wishes to improve the reproducibility of the work that we publish. This form provides structure for consistency and transparency in reporting. For further information on Nature Portfolio policies, see our Editorial Policies and the Editorial Policy Checklist.

Statistics

For all statistical analyses, confirm that the following items are present in the figure legend, table legend, main text, or Methods section.

n/a	Confirmed	
☐	☒	The exact sample size (*n*) for each experimental group/condition, given as a discrete number and unit of measurement
☐	☒	A statement on whether measurements were taken from distinct samples or whether the same sample was measured repeatedly
☐	☒	The statistical test(s) used AND whether they are one- or two-sided<br>*Only common tests should be described solely by name; describe more complex techniques in the Methods section.*
☒	☐	A description of all covariates tested
☒	☐	A description of any assumptions or corrections, such as tests of normality and adjustment for multiple comparisons
☐	☒	A full description of the statistical parameters including central tendency (e.g. means) or other basic estimates (e.g. regression coefficient) AND variation (e.g. standard deviation) or associated estimates of uncertainty (e.g. confidence intervals)
☐	☒	For null hypothesis testing, the test statistic (e.g. *F*, *t*, *r*) with confidence intervals, effect sizes, degrees of freedom and *P* value noted<br>*Give P values as exact values whenever suitable.*
☒	☐	For Bayesian analysis, information on the choice of priors and Markov chain Monte Carlo settings
☒	☐	For hierarchical and complex designs, identification of the appropriate level for tests and full reporting of outcomes
☒	☐	Estimates of effect sizes (e.g. Cohen's *d*, Pearson's *r*), indicating how they were calculated

Our web collection on statistics for biologists contains articles on many of the points above.

Software and code

Policy information about availability of computer code

Data collection

- Western blots were scanned using the Image Lab Touch software (v2.3.0.07).
- ChIP-qPCR data were collected using the LightCycler480 software (v1.5.1.62) or the built-in CFX384 Touch software (version number not provided).
- Nano-C data were collected using the MinKNOW software (latest version available at the time of the experiments).
- Cryo-EM data were collected using the Super Coolscan software (version number not provided).
- DNA-FISH data were collected using the LAS X Software Platform (v3.3).

Data analysis

- ChIP-qPCR data were analyzed using Microsoft Excel (v16.75.2).
- ChIP-seq data were mapped to the ENSEMBL Mouse genome assembly GRCm38.p6 (mm10) using BWA (v0.7.15). Peaks were identified with MACS2 (v2.1.1.20160309). CTCF motif analysis was done using the MEME-suite (v5.0.2).
- SLIM-ChIP data were mapped to the ENSEMBL Mouse genome assembly GRCm38.p6 (mm10) using bowtie2 (v2.4.2). reads were filtered using SAMtools (v1.10). Genome-wide coverage was determined using the BEDtools suite (v2.30.0). Peaks were identified with MACS2 (v2.2.7.1).
- Hi-C data were mapped to the ENSEMBL Mouse genome assembly GRCm38.p6 (mm10) and processed using HiC-Pro (v2.9.0) and Bowtie2 (v2.3.0). TAD boundaries were called using TADtool (v0.76). Intersection with CTCF ChIP-seq datas was done using the BEDtools suite (v2.26.0).
- 4C-seq data were mapped to the ENSEMBL Mouse genome assembly GRCm38.p6 (mm10) and processed using the c4ctus tool (unreleased version available from https://github.com/NoordermeerLab/c4ctus).
- Nano-C data were basecalled using Guppy (v4.0.11). Reads were mapped to viewpoints using BWA-MEM (v0.7.15), followed by filtering using the MinIONQC tool (v1.4.2). Retained reads were mapped to the repeat-masked ENSEMBL Mouse genome assembly GRCm38.p6 (mm10) using BWA-MEM (v0.7.15), followed by filtering using the MinIONQC tool (v1.4.2). Further Nano-C analysis was performed using in-

house code (unreleased version available from https://github.com/NoordermeerLab/Nano-C).
- Cryo-EM data were analysed using ImageJ (v1.53).
- RNA-seq data were mapped to the ENSEMBL Mouse assembly GRCm38.p6 (mm10) using STAR (v2.4.2a).
- DNA-FISH images were deconvolved with Huygens Essential (v18.10). Images were analyzed using the TANGO plug-in for ImageJ (v0.97).

For manuscripts utilizing custom algorithms or software that are central to the research but not yet described in published literature, software must be made available to editors and reviewers. We strongly encourage code deposition in a community repository (e.g. GitHub). See the Nature Portfolio guidelines for submitting code & software for further information.

Data

Policy information about availability of data

All manuscripts must include a data availability statement. This statement should provide the following information, where applicable:

- Accession codes, unique identifiers, or web links for publicly available datasets
- A description of any restrictions on data availability
- For clinical datasets or third party data, please ensure that the statement adheres to our policy

Rad21 ChIP-seq data were obtained from the GEO repository [GSE33346 (https://www.ncbi.nlm.nih.gov/geo/query/acc.cgi?acc=GSE33346)].
CTCF SLIM-ChIP data were obtained from the GEO repository [GSE108948 (https://www.ncbi.nlm.nih.gov/geo/query/acc.cgi?acc=GSE108948)].
Hi-C data were obtained from the GEO repository [GSE96107 (https://www.ncbi.nlm.nih.gov/geo/query/acc.cgi?acc=GSE96107)].

The unprocessed Oxford Nanopore Technologies and Illumina sequencing data generated in this study (Nano-C, ChIP-seq, 4C-seq, RNA-seq) have been deposited in the European Nucleotide Archive (EMBL-EBI ENA) database under accession code PRJEB44135 (https://www.ebi.ac.uk/ena/browser/view/PRJEB44135). The processed sequencing data (Nano-C, ChIP-seq, 4C-seq, RNA-seq) and Cryo-EM images are available at the Mendeley Data repository under accession code 10.17632/g7b4z8957z.4 (https://data.mendeley.com/datasets/g7b4z8957z/4). Unprocessed and processed data are available without restrictions.

Research involving human participants, their data, or biological material

Policy information about studies with human participants or human data. See also policy information about sex, gender (identity/presentation), and sexual orientation and race, ethnicity and racism.

Reporting on sex and gender	-
Reporting on race, ethnicity, or other socially relevant groupings	-
Population characteristics	-
Recruitment	-
Ethics oversight	-

Note that full information on the approval of the study protocol must also be provided in the manuscript.

Field-specific reporting

Please select the one below that is the best fit for your research. If you are not sure, read the appropriate sections before making your selection.

☒ Life sciences ☐ Behavioural & social sciences ☐ Ecological, evolutionary & environmental sciences

For a reference copy of the document with all sections, see nature.com/documents/nr-reporting-summary-flat.pdf

Life sciences study design

All studies must disclose on these points even when the disclosure is negative.

Sample size	Genomics experiments used for analysis and the ChIP-qPCR experiments were performed in at least 2 biological duplicates (see Methods section for detail). Experiments for visualization or validation (Western blotting, 4C-seq, Cryo-EM, DNA-FISH, and the CTCF ChIP-seq in CBS 20326 and CTCF-AID cells) were performed once.
Data exclusions	For ChIP-qPCR, individual data points with low quality melting curves were excluded. For all other experiments, no data was excluded.
Replication	All genomics experiments were performed on at least 2 biological replicates, with the exception of CTCF ChIP-seq in CBS 20326 and CTCF-AID cells. Genomics data sets were validated by visual observations, followed by merging for downstream analysis and visualization. Imaging and Cryo-EM were not replicated.
Randomization	The software-based analysis of the data is not compatible with randomization.
Blinding	The software-based analysis of the data is not compatible with blinding.

Reporting for specific materials, systems and methods

We require information from authors about some types of materials, experimental systems and methods used in many studies. Here, indicate whether each material, system or method listed is relevant to your study. If you are not sure if a list item applies to your research, read the appropriate section before selecting a response.

Materials & experimental systems

n/a	Involved in the study
☐	☒ Antibodies
☐	☒ Eukaryotic cell lines
☒	☐ Palaeontology and archaeology
☒	☐ Animals and other organisms
☒	☐ Clinical data
☒	☐ Dual use research of concern
☒	☐ Plants

Methods

n/a	Involved in the study
☐	☒ ChIP-seq
☒	☐ Flow cytometry
☒	☐ MRI-based neuroimaging

Antibodies

Antibodies used

CTCF antibody (07-729, Merck-Millipore)
Rad21 antibody (ab992, Abcam)
Lamin-B1 antibody (ab65986, Abcam)
H3K4me3 antibody (07-473, Merck-Millipore)
H3K27ac antibody (39133, Active Motif)
H3K27me3 antibody (17-622, Merck-Millipore)
H3K36me3 antibody (ab9050, Abcam)

For Western blotting, the following dilutions were used: 1:500 dilution of CTCF antibody, 1:1000 dilution of Rad21 antibody and 1:500 dilution of Lamin B1 antibody.

For ChIP, the following dilutions were used for 10 µg of chromatin: 5 µg CTCF antibody, 2 µl H3K4me3 antibody, 5 µg H3K27ac antibody, 5 µg H3K27me3 antibody, 4 µg H3K36me3 antibody.

Validation

All antibodies were validated by their suppliers using Western blotting. The H3K4me3 antibody (07-473, Merck-Millipore), H3K27ac antibody (39133, Active Motif), H3K27me3 antibody (17-622, Merck-Millipore) and H3K36me3 antibody (ab9050, Abcam) were validated by ChIP-qPCR by the supplier.

No further validations were performed.

Eukaryotic cell lines

Policy information about cell lines and Sex and Gender in Research

Cell line source(s)

All cell lines are mouse embryonic stem cells: E14Tg2a.4 or their derivatives.
The E14Tg2a.4 mESC line was a gift from Joke van Bemmel and Edith Heard (Institut Curie, Paris, France) (Van Bemmel et al, 2019).
The CTCF-AID line was a gift from Elphège P. Nora (UCSF, San Francisco, USA), Benoit Bruneau (Gladstone Institutes, San Francisco, USA) and Maxim Greenberg (Institut Jacques Monod, Paris, France) (Nora et al, 2017).
The Rad21-AID line was a gift from Qing Ling Liu and Elzo de Wit (Dutch Cancer Institute, Amsterdam, The Netherlands) (Liu et al, 2021).

Authentication

Cell lines were not authenticated, beyond their capacity for proliferation and morphology in the dedicated embryonic stem cell medium.

Degradation of CTCF and Rad21 upon Auxin treatment was confirmed using Western blotting.

CRISPR-Cas9 modifications of CBS 20326 were confirmed by PCR, followed by Sanger sequencing of at least 8 independent clones obtained after ligation of each PCR band into the pGEM-T system.

Mycoplasma contamination

All cell lines were regularly validated to be negative for mycoplasma contamination.

Commonly misidentified lines
(See ICLAC register)

No commonly misidentified cell lines were used in the study.

Plants

Seed stocks

-

Novel plant genotypes	-
Authentication	-

ChIP-seq

Data deposition

☒ Confirm that both raw and final processed data have been deposited in a public database such as GEO.

☒ Confirm that you have deposited or provided access to graph files (e.g. BED files) for the called peaks.

Data access links<br>*May remain private before publication.*	Raw data: https://www.ebi.ac.uk/ena/browser/view/PRJEB44135<br>Processed data: https://data.mendeley.com/datasets/g7b4z8957z/4
Files in database submission	Raw data:<br>ftp.sra.ebi.ac.uk/vol1/run/ERR571/ERR5718554/mES.CTCF.20150422.20151012.fastq.gz<br>ftp.sra.ebi.ac.uk/vol1/run/ERR571/ERR5718555/mES.CTCF.20150915.20151102.fastq.gz<br>ftp.sra.ebi.ac.uk/vol1/run/ERR571/ERR5718556/mES.CTCF.20150915.20151207.fastq.gz<br>ftp.sra.ebi.ac.uk/vol1/run/ERR571/ERR5718557/mES.CTCF.20150915.20160120.fastq.gz<br>ftp.sra.ebi.ac.uk/vol1/run/ERR571/ERR5718558/mES.H3K27ac.20150422.20161114.fastq.gz<br>ftp.sra.ebi.ac.uk/vol1/run/ERR571/ERR5718559/mES.H3K27ac.20150915.20160908.fastq.gz<br>ftp.sra.ebi.ac.uk/vol1/run/ERR571/ERR5718560/mES.H3K27me3.20150422.20150724.fastq.gz<br>ftp.sra.ebi.ac.uk/vol1/run/ERR571/ERR5718561/mES.H3K27me3.20150428.20150921.fastq.gz<br>ftp.sra.ebi.ac.uk/vol1/run/ERR571/ERR5718562/mES.H3K36me3.20150422.20151012.fastq.gz<br>ftp.sra.ebi.ac.uk/vol1/run/ERR571/ERR5718563/mES.H3K36me3.20150428.20150921.fastq.gz<br>ftp.sra.ebi.ac.uk/vol1/run/ERR571/ERR5718564/mES.H3K4me3.20150422.20151012.fastq.gz<br>ftp.sra.ebi.ac.uk/vol1/run/ERR571/ERR5718565/mES.H3K4me3.20150428.20150921.fastq.gz<br>ftp.sra.ebi.ac.uk/vol1/run/ERR571/ERR5718566/mES.Input.20150422.20150921.fastq.gz<br>ftp.sra.ebi.ac.uk/vol1/run/ERR571/ERR5718567/mES.Input.20150422.20150724.fastq.gz<br>ftp.sra.ebi.ac.uk/vol1/run/ERR571/ERR5718568/mES.Input.20150915.20160310.fastq.gz<br>ftp.sra.ebi.ac.uk/vol1/run/ERR571/ERR5718569/CTCFcontrol.CTCF.fastq.gz<br>ftp.sra.ebi.ac.uk/vol1/run/ERR571/ERR5718571/CTCF-AID.CTCF.fastq.gz
Genome browser session<br>(e.g. UCSC)	No longer applicable.

Methodology

Replicates	All ChIP-seq experiments were performed in at least 2 biological duplicates, with the exception of the CTCF ChIP-seq experiments in CTCF-AID and CTCF CBS 20326 cells and their controls (data used only for visualization).
Sequencing depth	All sequencing experiments were performed in SE mode, with a minimum read length of 50 bp. The raw number of reads for each data set is provided below:<br>ftp.sra.ebi.ac.uk/vol1/run/ERR571/ERR5718554/mES.CTCF.20150422.20151012.fastq.gz 59240637<br>ftp.sra.ebi.ac.uk/vol1/run/ERR571/ERR5718555/mES.CTCF.20150915.20151102.fastq.gz 15162995<br>ftp.sra.ebi.ac.uk/vol1/run/ERR571/ERR5718556/mES.CTCF.20150915.20151207.fastq.gz 31373902<br>ftp.sra.ebi.ac.uk/vol1/run/ERR571/ERR5718557/mES.CTCF.20150915.20160120.fastq.gz 91002779<br>ftp.sra.ebi.ac.uk/vol1/run/ERR571/ERR5718558/mES.H3K27ac.20150422.20161114.fastq.gz 46677599<br>ftp.sra.ebi.ac.uk/vol1/run/ERR571/ERR5718559/mES.H3K27ac.20150915.20160908.fastq.gz 48264176<br>ftp.sra.ebi.ac.uk/vol1/run/ERR571/ERR5718560/mES.H3K27me3.20150422.20150724.fastq.gz 104824555<br>ftp.sra.ebi.ac.uk/vol1/run/ERR571/ERR5718561/mES.H3K27me3.20150428.20150921.fastq.gz 86997974<br>ftp.sra.ebi.ac.uk/vol1/run/ERR571/ERR5718562/mES.H3K36me3.20150422.20151012.fastq.gz 150264437<br>ftp.sra.ebi.ac.uk/vol1/run/ERR571/ERR5718563/mES.H3K36me3.20150428.20150921.fastq.gz 77352761<br>ftp.sra.ebi.ac.uk/vol1/run/ERR571/ERR5718564/mES.H3K4me3.20150422.20151012.fastq.gz 62024708<br>ftp.sra.ebi.ac.uk/vol1/run/ERR571/ERR5718565/mES.H3K4me3.20150428.20150921.fastq.gz 79117287<br>ftp.sra.ebi.ac.uk/vol1/run/ERR571/ERR5718566/mES.Input.20150422.20150921.fastq.gz 91918830<br>ftp.sra.ebi.ac.uk/vol1/run/ERR571/ERR5718567/mES.Input.20150422.20150724.fastq.gz 101105169<br>ftp.sra.ebi.ac.uk/vol1/run/ERR571/ERR5718568/mES.Input.20150915.20160310.fastq.gz 93232432<br>ftp.sra.ebi.ac.uk/vol1/run/ERR571/ERR5718569/CTCFcontrol.CTCF.fastq.gz 33649156<br>ftp.sra.ebi.ac.uk/vol1/run/ERR571/ERR5718571/CTCF-AID.CTCF.fastq.gz 41102361
Antibodies	CTCF antibody (07-729, Merck-Millipore)<br>H3K4me3 antibody (07-473, Merck-Millipore)<br>H3K27ac antibody (39133, Active Motif)<br>H3K27me3 antibody (17-622, Merck-Millipore)<br>H3K36me3 antibody (ab9050, Abcam)
Peak calling parameters	CTCF binding peaks were identified from the combined biological and technical replicates with MACS2 (v2.1.1.20160309).
Data quality	Duplicate reads, reads with multiple alignments and low-quality reads were removed, followed by the calculation of densities for combined biological and technical replicates

Software	BWA (v0.7.15), MACS2 (v2.1.1.20160309), MEME-suite (v5.0.2)
